# Barriers to Mental Health Treatment Utilization in Wards 7 and 8 in Washington, DC: A Qualitative Pilot Study

**DOI:** 10.1089/heq.2017.0051

**Published:** 2018-08-01

**Authors:** Ollie Ganz, Laurel E. Curry, Paulette Jones, Katherine H. Mead, Monique M. Turner

**Affiliations:** ^1^ Department of Prevention and Community Health, Milken Institute School of Public Health, George Washington University, Washington, District of Columbia.; ^2^ Center for Health Policy Science and Tobacco Research, RTI International, Washington, District of Columbia.; ^3^ Paving the Way Multi-Service Institute, Washington, District of Columbia.; ^4^ Department of Health Policy and Management, Milken Institute School of Public Health, George Washington University, Washington, District of Columbia.

**Keywords:** mental health, mental health treatment utilization, urban health

## Abstract

**Purpose:** There is a paucity of data on barriers to mental health treatment utilization among residents of Wards 7 and 8 in Washington, DC, despite exposure to many environmental factors that are associated with poor mental health outcomes and the high prevalence of mental health problems among residents. The objective of this study was to examine barriers to mental healthcare utilization among residents of Wards 7 and 8.

**Methods:** This study included semi-structured, in-depth interviews with five key informants who lived or spent significant time in Wards 7 or 8 in Washington, DC, which are the wards served by Paving the Way MSI, a behavioral health clinic that served as a partner organization in the study.

**Results:** Barriers to mental health treatment utilization existed at a variety of social-ecological levels, including the individual/interpersonal level, the provider/mental health system level, the community level, and the societal level. Major barriers included fear and trust/distrust in the medical system, lack of social support, the model of mental healthcare, lack of patient-centered care, limited access to mental health services, stigma of mental illness and mental health treatment, and poverty.

**Conclusion:** This study highlights the need to address barriers to mental health treatment utilization at multiple social-ecological levels. Future studies should examine perspectives from residents with mental health problems in these wards to gain a more thorough understanding of the barriers to treatment. Funding is needed to support efforts to increase mental health treatment utilization among residents of Wards 7 and 8.

## Introduction

Among low-income individuals and African Americans, utilization of mental health services is low.^1,2^ African Americans are less likely than whites to receive mental healthcare and treatment^2–6^ due to a variety of barriers, including low rates of insurance coverage,^7^ lack of a regular healthcare provider,^7^ difficulty finding resources for treatment of mental health problems,^8^ distrust in the healthcare system,^9^ competing health demands,^10^ and stigma of mental health and mental health treatment.^9,11–13^

Mirroring this national trend, adults living in Wards 7 and 8 in Washington, DC, who are predominately low-income and African American,^14,15^ experience higher rates of mental health problems compared with the rest of the District.^14–17^ This part of Washington, DC is emblematic of distressed, isolated, and historically disadvantaged communities in cities across the United States.^18^ This area has long been underserved, with resources disproportionately invested west of the river.^19,20^ Many environmental factors that are associated with poor mental health outcomes, including experiences with discrimination, violence, reduced access to health services, and poverty,^21^ disproportionately affect individuals in these Wards.^22–28^ Further, Wards 7 and 8 are medically underserved areas with a shortage of healthcare providers, including mental health providers.^17^

Despite this area's history as an underserved part of Washington, DC, and disproportionate rates of mental health issues among its residents, factors related to treatment utilization specific to individuals living in Wards 7 and 8 have gone unexamined. Understanding mental health treatment utilization among this population is key to developing efforts to facilitate mental health treatment in urban neighborhoods that are particularly vulnerable to mental health problems.

## Methods

### Sample and study procedures

Sample size was limited to five individuals since funding for this pilot study was very limited. Inclusion criteria required that participants be 18 years of age or greater and live or spend time in Wards 7 and 8. Participants were recruited through a partner organization, Paving the Way Multi Service Institute (PTW), a behavioral health clinic in Washington, DC. The staff at PTW identified potential participants who work in the mental health field, such as therapists, physicians, and employees at mental health advocacy organizations, who could serve as key informants (*n*=5) and could provide unique and specialized knowledge about mental health issues in Wards 7 and 8. To ensure that potential respondents understood that they could decline participation, study staff emphasized that their participation in the study was voluntary and that it would not affect the individual's relationship with Paving the Way.

The study consisted of semi-structured in-depth interviews, which occurred in March 2016. A gatekeeper at PTW recruited participants. In-depth interviews took place at PTW (Ward 8), since it was conveniently located for study participants. The main domains addressed in the interviews can be found in Table 1. This study was approved by the George Washington University Institutional Review Board.

**Table 1.** **Key Constructs Addressed in Qualitative In-Depth Interviews**

Social norms related to mental illness and mental health treatment
Mental health literacy
Barriers to mental health treatment utilization
Stigma of mental illness and mental health treatment
Access to mental healthcare
Contextual factors (e.g., poverty, incarceration)

### Analytic plan

Thematic content analysis was conducted to examine key themes related to barriers to mental health treatment utilization across interviews and to summarize similarities and differences within the data.^29,30^ Interviews were transcribed and then uploaded to NVivo 11.^31^ An integrated data analysis approach was used, which incorporated both inductive and deductive methods.^29,32^ Interviews were coded deductively by using a coding scheme based on the study's interview guide (Table 1).

## Results

Although the objective of this study was to examine factors that hinder and increase mental health treatment utilization, participants also provided insight into potential facilitators of mental health treatment utilization. The themes were categorized into the following groups: (1) societal-level factors, (2) community-level factors, (3) provider and medical system-related factors, and (4) individual and interpersonal factors. See Table 2 for illustrative quotes from each theme.

**Table 2.** **Barriers to Mental Health Treatment Utilization: Illustrative Quotes**

Theme	
Individual and Interpersonal-Level Factors	
Disconnect Between Symptoms and Treatment	“Being depressed is just a mood. It's like, I'm happy, I'm sad, I'm depressed. It's a mood. It doesn't necessarily ignite ‘I need help.’”
Fear and Distrust in the Medical System	“Well, it's trust and rapport, relationship, all this is kind of tied in. But there's a lot of distrust with government kind of stuff, institutions in general.”
Social and Familial Support	“Let's say your loved one has conveyed to the clinician what their triggers are but you see this person getting triggered, you just don't know that's what they're doing. Or, if they're being triggered and you don't know how to help them cope because you can't talk to the clinician.”
Triggering Experiences	“Most of my patients, the young ones, of course, the parents would bring him in. I'm finding I have now some people aging out now. 18, 19 year olds. They are coming on their own. They know what they need and they are coming. Once they've had a positive experience. Okay, so the challenge is getting in, and so they can experience what it is as opposed to some of the myths that they hear or some of the negative peer pressure they may get.”
Provider and Medical System-Related Factors	
Model of Mental Healthcare	“You have an ailment, you go to the doctor, the doctor prescribes you with some medicine. It's done, you're better, it's all good. So, there's a finality to whatever the symptom is, and I think that that's the difference between the mental health, is this is ongoing. This is an ongoing need.”
Patient-Centered Care	“If clinicians yielded more opportunity for feedback from consumers, I'm quite sure there would be a shift in stigma and perceptions and all this other good stuff that is, I feel, overshadowing the level of care that's being delivered.”
Community-Level Factors	
Access to Mental Health Services	“I've had a couple people that do live east of the river and when I ask them… do they want a new provider…I would say the majority of the time they will say no… Do you know what the reason is when they say no? Because they don't want to go that far. They don't want to go away to Georgetown. They don't want to go away to Sibley Hospital. They don't want to go, you know. They want to get their treatment near their home! But isn't that the case for the rest of us?”
Stigma	“They were the throwaways, the rejects, and the embarrassments, you know.”
Societal-Level Factors	
Poverty	“You're in flux, so you're always in crisis. Where a crisis is usually defined by a time…is time limited, but here you're talking about people that are always in flux/crisis. There's always a lack of money. There is always something going on in their lives.”

### Societal-level factors

Only one societal-level factor emerged from the data.

#### Poverty

Respondents described those living in poverty as having competing priorities and demands due to their living conditions. For example, many residents are dealing with housing instability, violence, and unemployment; therefore, mental health treatment may be a low priority or may be unaffordable. Participants described poverty as both a causal factor related to mental health problems and a barrier to mental health treatment utilization.

Respondents also described competing priorities in terms of comorbid conditions. For example, some residents may need to balance multiple health conditions at once and due to limited time and financial resources, mental health may not be a priority.

### Community-level factors

Two community-level factors emerged from the data and are detailed next.

#### Access to mental health services

Geographic access to mental health providers and facilities is a tangible barrier to mental health treatment in Wards 7 and 8. Although residents in Wards 7 and 8 could travel to other wards for treatment, many do not want to or are unable to due to other commitments and priorities. Further, if a patient requires regular treatment (i.e., weekly), a long commute to the mental health provider may not be sustainable, realistic, or affordable. Some patients may already be hesitant to get mental health treatment and a long commute would prevent them from doing it.

#### Stigma

Stigma of mental illness and mental health treatment emerged as a barrier to utilization of mental health services. Respondents described stigma as the perception that people with mental illness are “crazy.” Many residents perceive mental illness as only including extreme conditions, such as schizophrenia and drug addiction, and do not think about conditions such as depression and anxiety as being types of mental illness. Further, those who seek mental health treatment may be perceived as “weak.” They may be talked about as needing their “behind kicked” or needing to pray to be healed.

Participants discussed how the historical perspective of mental illness may be related to the stigma that many hold today. One participant talked about how in the 1960s and 1970s, people with mental illness were either integrated into society and their mental illness was hidden, or if that was not possible, they were sent away and institutionalized. This participant noted: “They were the throwaways, the rejects, and the embarrassments, you know.” This participant spoke about how this perspective can get passed down from one generation to another.

Several respondents noted that men appear to stigmatize mental illness and mental health treatment more than women. Further, participants noted that men were more resistant to mental healthcare and held stronger negative beliefs about those with mental illness, whereas women had fewer negative perceptions and were more willing to seek mental health treatment, particularly for their children.

The lack of mental health services in Wards 7 and 8 described earlier could be exacerbating stigma. The paucity of mental health providers gives the appearance that mental illness is not prevalent or normative. One participant brought up that improving access to mental health services in these Wards would not only likely increase utilization of mental health services but also make mental health treatment seem more normal and prevalent, and, thereby reduce stigma. Another potential solution brought up by a participant would be to incorporate mental health services into more primary care offices. This could potentially reduce stigma associated with seeking a mental health provider, since the individual would simply be going to the doctor, not to a mental health facility.

Study respondents described the effects of stigma of mental illness and mental health treatment on treatment seeking behavior. Specifically, respondents described that many individuals with mental health issues, as well as family members of those who suffer, experience feelings of shame, guilt, and embarrassment, and thus are hesitant to seek treatment.

Religion emerged as both a potential source and solution to stigma of mental illness and mental health treatment. Several participants stated that religious institutions could be a source of stigma, since mental illness may be ignored and treatment seeking may be perceived as spiritually weak. One participant noted that she was told by a member of a local church that her mental health work “conflicted with the teachings of the church, and the teachings of the church were that you basically pray it away.” On the other hand, participants stated that incorporating mental health services into religious institutions can also help to ameliorate distrust in and reduce stigma of mental health treatment.

### Provider and medical system-related factors

A total of two themes emerged related to provider and medical system-related factors.

#### Model of mental healthcare

The longitudinal model of mental healthcare emerged as a barrier to mental healthcare utilization. Participants discussed how many people are used to a model of care where one goes to the doctor when they have symptoms, they receive a diagnosis and are then prescribed medication to treat the ailment. In contrast, participants described mental healthcare as being an ongoing process, which they may not be familiar or comfortable with.

Requiring annual or biannual mental health screenings emerged as a potential solution to this barrier. Multiple respondents discussed how mental health screenings should be viewed like an annual physical, as a way to check in with a professional regarding the state of your mental health. One participant suggested giving a brief mental health screening to every patient who enters the emergency room, allowing for a potential mental health need to be recognized and addressed.

#### Patient-centered care

Patient-centered care came up as a potential facilitator of utilization of mental health services in Wards 7 and 8. This includes empowering patients with mental health problems to make choices about their care and treatment. One participant articulated the need for mental health providers to provide patients with options in terms of their treatment or to listen to or incorporate patient feedback into their treatment plans.

### Individual and interpersonal-level factors

A total of four themes emerged related to individual and interpersonal-level factors and are described next.

#### Disconnect between symptoms and treatment

Participants described how residents in Wards 7 and 8 experience high levels of stress, depression, and other mental health problems, but do not necessarily associate these symptoms with a mental health issue and do not think about mental health treatment as a solution. Participants described how many residents may acknowledge certain feelings or difficulties but do not necessarily view them as chronic issues. As one participant stated: “Being depressed is just a mood. It's like, I'm happy, I'm sad, I'm depressed. It's a mood. It doesn't necessarily ignite ‘I need help.’”

#### Fear and trust/distrust in the medical system

Several participants stated that some residents associate mental health treatment with medication that will change or control them. On the other hand, trust also emerged as a potential facilitator of use of mental health services. One participant, who is a health professional, described patient–provider trust as the key to continuity in care and successful treatment of mental health issues. This includes being nonjudgmental and actively listening to a patient's needs. This participant stated: “Well, it's trust and rapport, relationship, all this is kind of tied in.” The church was discussed as a setting where established trust can help to reduce barriers to utilization of mental health services. For example, one participant suggested that housing mental health services within a church could help to foster trust.

#### Social and familial support

The concept of familial support emerged as a facilitator of seeking treatment for mental health issues. One participant emphasized how “a person can't do it alone” and that individuals need support to get them into treatment and to also continue treatment, whether that be therapy and/or taking medication. One respondent addressed the need for mental health providers to coordinate with family members with regard to the patient's care to facilitate treatment adherence.

The idea of community support came up as well, specifically within the context of the church. One participant talked about the importance of the church speaking openly about mental health and mental health treatment and providing support to those in need. Participants also discussed the value of mental health services, such as Paving the Way, being housed within the church. This both symbolically and literally provides support to those suffering from mental health issues.

#### Triggering events

Triggers or life events that encourage someone to seek care emerged as facilitators to treatment utilization. One trigger that emerged was incarceration. One participant talked about how experiences with the criminal justice system expose some individuals to mental health treatment who would not have been otherwise.

Triggers can also include witnessing others having a positive experience with the mental health system. One participant noted that individuals may experience this when taking their children to receive treatment. If they see their child receiving comprehensive care from a psychiatrist and a school social worker, for example, and start to see success with their child from treatment, they may become more open to the idea of mental health treatment for themselves. Having a positive experience themselves also triggers some individuals to continue seeking care and to seek treatment again if needed.

## Discussion

This study demonstrates that barriers to mental health treatment utilization in Wards 7 and 8 exist at a variety of social-ecological levels, including the individual/interpersonal level, the provider/mental health system level, the community level, and the societal level. To our knowledge, this is the first study to provide insight into barriers and facilitators that are specific to Wards 7 and 8 in Washington, DC. However, many themes that emerged are consistent with other studies, including the role of stigma as a barrier to treatment^12^ and the impact that negative experiences with and distrust of the mental healthcare system can have on use of services.^33^

In any examination of mental health treatment utilization, mental health itself must be included, as these two issues are intertwined. The profound impact of poverty emerged as a dominant factor influencing both mental health and treatment utilization among residents of Wards 7 and 8, as the effects of poverty can cause stress, depression, and other negative mental health symptoms, while also limiting one's ability to use mental health services. Respondents spoke at length about residents being entrenched in poverty, experiencing homelessness, unemployment, violence, and trauma, among many other devastating consequences of poverty. Research demonstrates that poverty and mental health are a vicious cycle, with poverty causing mental illness and mental illness also being a cause of poverty.^27^ Future research and interventions designed to address the underutilization of mental health services must address and examine the role of poverty as both a cause of mental health problems and a barrier to mental health treatment.

Various individual and community-level factors emerged as barriers to treatment utilization. For example, stigma of mental health treatment utilization was described as a barrier to receiving treatment. To overcome this, several participants suggested integrating mental health treatment services into venues that residents already frequent. For example, integrating mental health services into primary care or housing providers within a church, which is how Paving the Way is structured, makes it easier for those in need to seek help. Studies have shown that integrating mental health services into the primary care setting and church environment can be effective in reducing the mental health treatment gap.^34,35^

This study has limitations. First, the method of study recruitment may have introduced bias into the study. Participants who enrolled in the study were likely more comfortable discussing mental health issues than the average resident of Wards 7 and 8, therefore creating a bias. Further, the study sample size of 5 is smaller than what is standard in the field for in-depth interviews and, therefore, saturation was not reached in conducting the interviews. However, many overlapping themes still emerged from the data. In addition, participants were key informants and not necessarily residents themselves and, therefore, were one level removed from actual residents experiencing barriers to treatment. Since this study included key informants, next steps would be to interview residents of Wards 7 and 8, including both individuals who have received mental health treatment and those who have not, to get a variety of perspectives. Further, a quantitative assessment based on the qualitative research should be developed and implemented to assess both factors related to mental health problems and barriers to treatment utilization on a larger scale in Wards 7 and 8. An examination of the quality of healthcare across Wards is also needed to provide insight into the disproportionate prevalence of mental health problems in this area.

This study highlights the need to address factors at various levels when considering barriers to mental health treatment utilization, including individual-level factors, as well as the social determinants of health, such as housing instability and violence. In addition, there is a clear need for more mental health providers in Wards 7 and 8, as there were only six mental health facilities as of 2012.^17^ Further, a health communication campaign aimed at normalizing mental health problems and mental health treatment and reducing stigma could be useful in Wards 7 and 8. More funding is needed to support these efforts.

## Implications for Health Equity

Mental health problems have an enormous negative impact on one's quality of life, and the availability and utilization of mental health treatment is crucial for allowing those affected by mental health problems to lead fulfilling lives.^36^ Many mental health issues, including anxiety and depression, and risk factors for mental illness, disproportionately impact individuals living in Wards 7 and 8 in Washington, DC. Future research efforts can build upon these findings to inform clinical and policy interventions designed to achieve equitable access to and utilization of mental health treatment.

## Abbreviation Used

PTW: Paving the Way Multi Service Institute
